# Study on bearing capacity of Mortise-tenon and joint-flange concrete assembled foundation of transmission line under combined load

**DOI:** 10.1371/journal.pone.0327965

**Published:** 2025-08-01

**Authors:** Xin Hu, Xiaojuan Xi, Yuesong Zheng, Zijun Xiang, Han Zhang

**Affiliations:** 1 State Grid Henan Economic Research Institute, Zhengzhou, Henan Province, China; 2 College of Electrical Engineering and New Energy, China Three Gorges University, Yichang, Hubei, China; Macau University of Science and Technology, MACAO

## Abstract

Mortise-tenon and joint-flange assembled foundation has excellent application as a new type of slab concrete assembled foundation, but there is a lack of research on its bearing capacity. In order to explore the mechanical characteristics and bearing capacity of this type of foundation under combined load (uplift-horizontal load), which is different from the traditional cast-in-place foundation, the uplift bearing model of mortise-tenon and joint-flange assembled foundation and the uplift model of cast-in-place foundation with the same specification were established based on the actual geological environment by finite element software. The stress distribution, vertical and horizontal displacements, and uplift and horizontal bearing capacities of the foundations were simulated and calculated. This study found that the bearing capacity of the Mortise-tenon and joint-flange assembled foundation has not been fully utilized. Specifically, the deformation of the foundation mainly concentrates on the main column, and the load is unable to be transmitted to the lower structure through the flange. Under combined loading (uplift-horizontal load), the load-displacement relationship curve can be roughly divided into three stages: linear slow rise stage, plastic accelerated rise stage, and linear failure stage. During the pull-out process, the foundation demonstrates stress characteristics of segmented load transmission. After the concrete upper column yields, the mortise-tenon and joint-flange connection node receives the load transmitted by the upper column, and continues to transmit the load to the lower column of the foundation after the displacement of the node reaches its limit. When the uplift cumulative displacement of the foundation reaches approximately 13 mm and the horizontal cumulative displacement reaches around 10 mm, the foundation reaches its ultimate state. At this point, its ultimate bearing capacity surpasses that of the cast-in-place foundation of the same specification, with significant improvement. The ultimate uplift bearing capacity increases by 33.34%, while the ultimate horizontal bearing capacity increases by 48.09%.

## 1. Introduction

In Western China, the increasing demand for electricity and rapid development of transmission lines have highlighted the limitations of traditional cast-in-place foundations, such as high costs, long construction times, and environmental impacts [1–4]. In contrast, prefabricated foundations, made in factories and assembled on-site, offer significant advantages. They reduce labor and material costs, improve quality control, enhance mechanization, and lower construction difficulty [5]. As a result, prefabricated foundations are seen as a promising solution for transmission line projects in complex terrains [6]. For brevity, the term “MTJF assembled foundation” will hereafter be used to denote the mortise–tenon and joint–flange assembled foundation throughout the remainder of this paper.

Currently, while cast-in-place concrete foundations remain the most common, other types of transmission tower foundations are also used based on site conditions. Rock anchor foundations leverage the strength of rock masses, offering material savings and environmental benefits but require high-quality rock and detailed surveys, making them unsuitable for complex terrain [7]. Slab (raft) foundations distribute loads effectively but consume large amounts of concrete and face material transportation challenges [8]. Pile foundations reach deeper stable strata but rely on heavy machinery, limiting their use in difficult terrain [9]. In such environments, prefabricated modular foundations offer a more practical alternative due to easier transport, quicker installation, and better adaptability.

The application and advancement of assembled foundations in transmission lines are currently constrained by several technical challenges, including the fabrication complexity of irregular components, transportation difficulties due to excessive weight, intricate assembly at connection nodes, and low on-site installation efficiency [10]. To address these limitations, a novel assembled concrete foundation system has been developed, incorporating mortise–tenon and joint–flange connections. This system effectively reduces the size and quantity of components, streamlines connection procedures, and significantly improves installation efficiency while ensuring adequate load-bearing capacity [11]. During operation, the foundation is subjected to combined actions of self-weight and environmental loads such as wind and ice accretion, resulting in multidirectional stress distributions in both vertical and horizontal directions [12–13]. The foundation’s bearing performance is influenced by its structural configuration, embedment depth, and surrounding soil properties. Its ultimate bearing capacity is jointly governed by both the intrinsic strength of the foundation and the bearing characteristics of the subgrade. Under horizontal loading, bending moments are induced in the primary columns, further affecting structural performance. Therefore, the interaction of vertical and horizontal loads critically determines the stability and reliability of assembled foundations [14–15].

Zhu Zhaoqing [16], based on full-scale tests of assembled foundations, studied the bearing performance of concrete assembled foundations under uplift-horizontal combined loads. The results indicated that under combined loads, the bearing performance of multi-layer mortise-tenon structures is superior to that of single-layer mortise-tenon structures, and the component nodes of the assembled foundation fail before the components themselves. Qian Zengzhen and colleagues [17] conducted field experiments on metal-concrete assembled foundations in desert terrain, investigating their deformation characteristics and structural stress behavior under compression-horizontal combined loads. The study found that under ultimate compression-horizontal loading conditions, the foundation lost its bearing capacity due to instability of the angle steel brackets. Dong Yiyi and collaborators [18] conducted prototype uplift tests of all-metal assembled foundations in sandy terrain, summarizing their uplift bearing performance and the deformation characteristics of foundation components. The study employed the soil weight method to simplify the calculation of uplift bearing capacity. Based on experimental and numerical simulation results, recommended values for the uplift angle in the soil weight method were proposed.

The above studies primarily focus on the bearing performance of assembled foundations under unidirectional loads or combined loads in all-metal and metal-concrete assembled foundations. These findings indicate that traditional assembled foundations exhibit distinct bearing performance and deformation states under different directional loads. However, the deformation characteristics and associated deformation trends of metal–concrete prefabricated foundations remain insufficiently explored. In addition, the bearing performance and stress characteristics of mortise-tenon and joint-flange assembled foundations under combined loads still require further investigation. For a more comprehensive overview of the characteristics of each reference, please refer to Table 1.

**Table 1:** Comparison of references.

References	Authors	Foundation Forms	Advantages	Disadvantages
Reference [16]	Zhu Zhaoqing, Jin Li, She Zhibin, et al.	Prefabricated Concrete Foundation with Embedded Steel Pipes	Easy to assemble, simple to construct, and controllable quality of components	Insufficient assembly process, and difficulty in controlling on-site accuracy
Reference [17]	Qian Zengzhen, Lu Xianlong	Semi-metallic, semi-concrete prefabricated foundation	It has a certain bearing capacity while ensuring a short construction period.	Instability of metal components under extreme conditions.
Reference [18]	Dong Yiyi, Feng Heng, Zeng Erxian, et al.	Fully metallic prefabricated foundation	High material strength, strong stability, and short construction period	High cost, and geological corrosion reduces bearing capacity.

To address the aforementioned issues, this study draws on current research methods for concrete assembled foundations and uses finite element analysis software to simulate the bearing performance of mortise-tenon and joint-flange assembled foundations under vertical-horizontal combined load conditions. Based on this, a simulation model of cast-in-place concrete foundations of the same specifications is developed to study the deformation states and stress distribution characteristics that distinguish mortise-tenon and joint-flange assembled foundations from cast-in-situ foundations, summarizing the stress characteristics of the new assembled concrete foundation. Taking the mortise-tenon and joint-flange assembled foundation as the research object, the study explores the impact of combined loads on the foundation’s bearing capacity, providing a basis and reference for the future design, manufacturing, and application of such assembled foundations.

## 2. Numerical simulation method

### 2.1 Structural parameters of mortise-tenon and joint-flange assembled foundations for transmission lines

**Fig 1:**
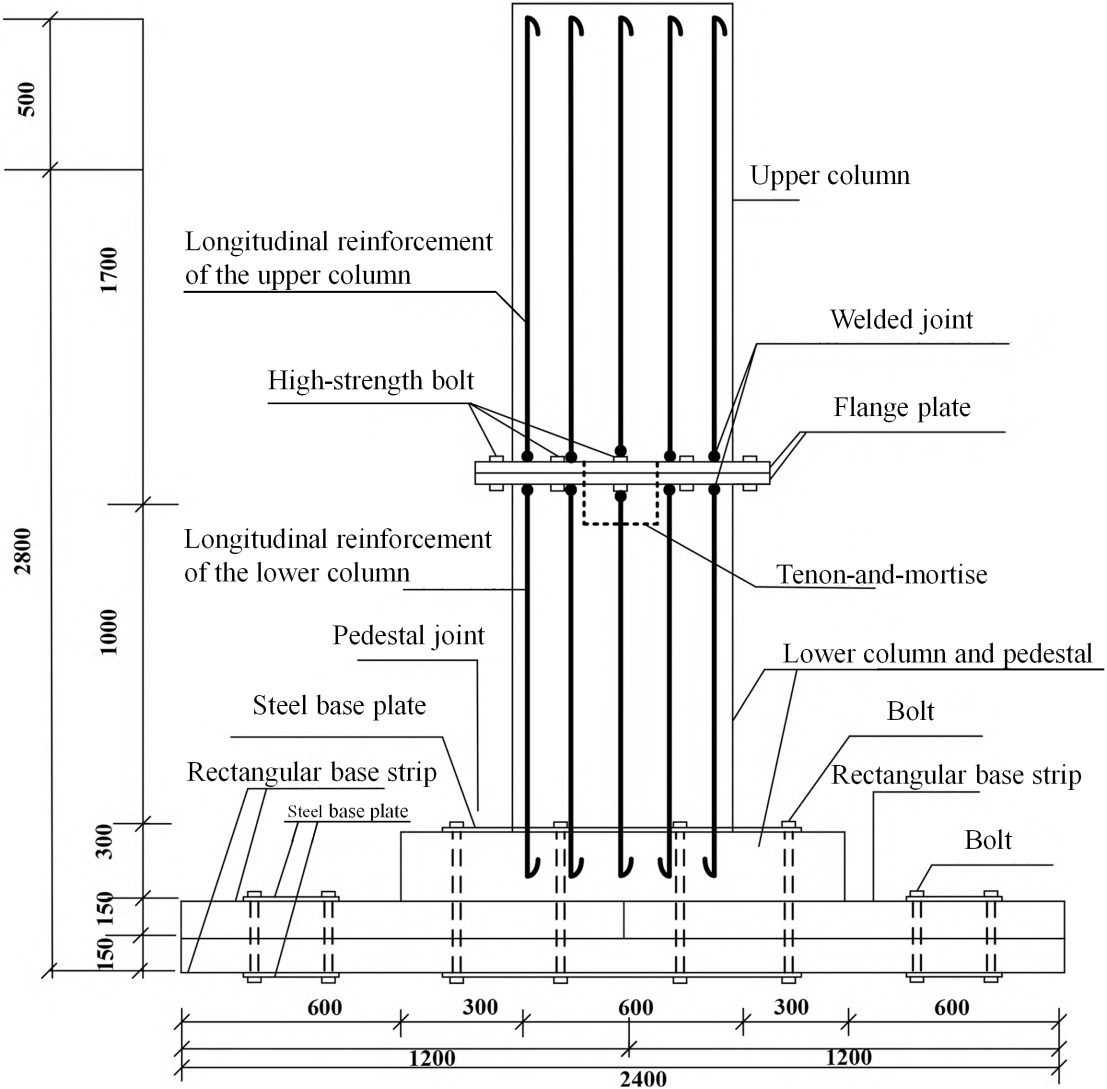
Specific structure of the new concrete assembled foundation.

The MTJF assembled foundation consists of multiple concrete assembled components connected through mortise-tenon and joint-flange. The modeled foundation has a total height of 3.2 m, a burial depth of 2.8 m, an upper concrete column dimension of 600 mm × 600 mm × 2200 mm, a lower concrete column dimension of 600 mm × 600 mm × 1000 mm, and a pedestal dimension of 1200 mm × 1200 mm × 300 mm. The concrete base plate consists of four rectangular base strips arranged in two layers and stacked vertically. The dimension of each rectangular base strip is 1200 mm × 1200 mm × 1500 mm. The primary foundation connections are as follows: the upper and lower columns are joined using a mortise-tenon and joint-flange connection, with 16 bolts symmetrically and evenly spaced around the joint-flange plate at intervals of 172.5 mm. The pedestal is bolted to the concrete base plate, with 12 bolts symmetrically and evenly spaced around the pedestal at intervals of 300 mm. The concrete base plate is composed of four identically sized rectangular base strips, which are also bolted together. Each base strip is equipped with 8 bolts, with a bolt spacing of 200 mm. The specific structure is shown in Fig 1.

**Fig 2:**
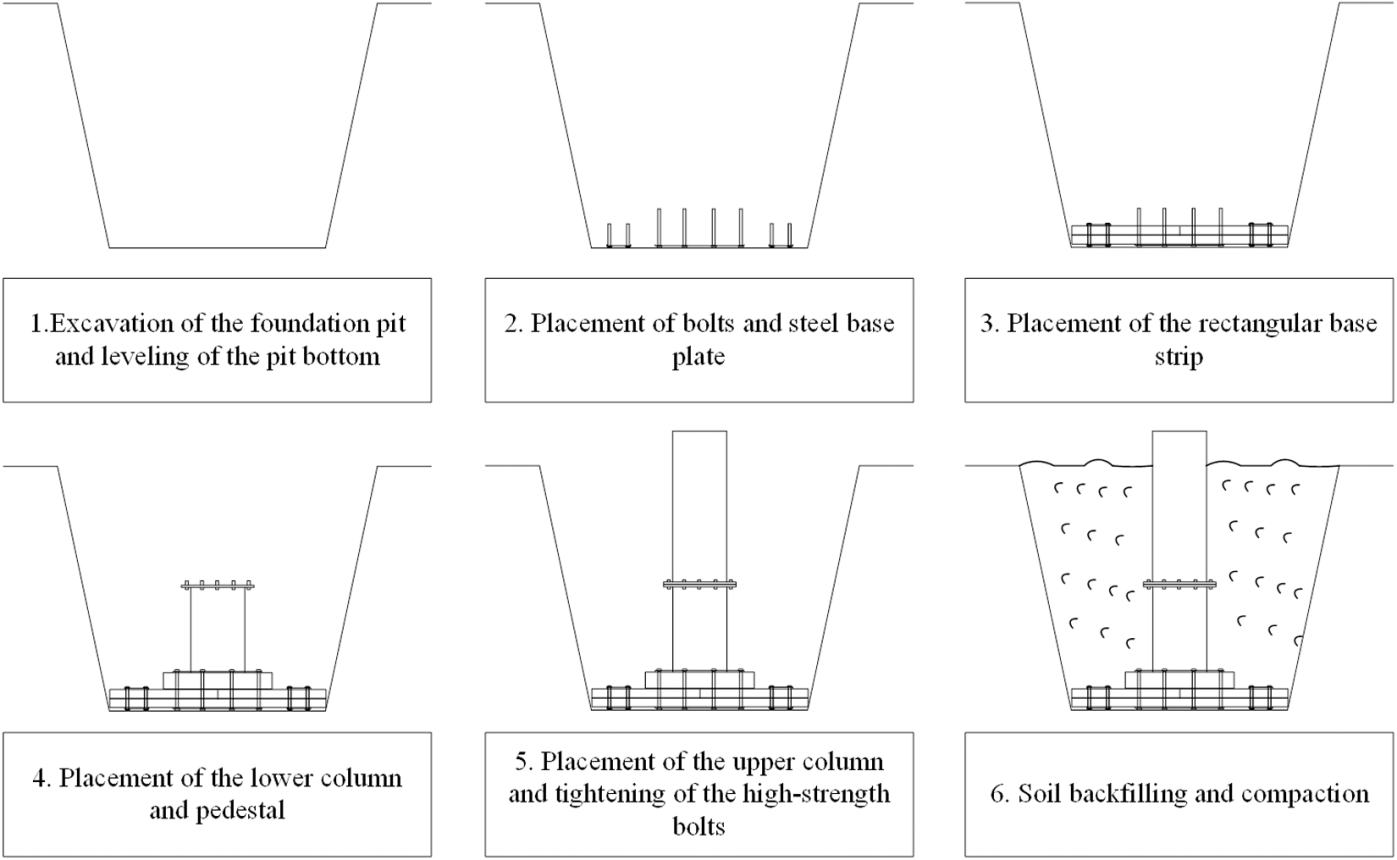
Simplified construction process of the proposed foundation.

Fig 2 presents the construction process flow for the proposed foundation. The process begins with the excavation of the foundation pit, followed by cleaning and leveling the pit bottom. Subsequently, bolts and the steel baseplate are positioned, ensuring their correct placement and spacing to align with the pre-marked holes in the rectangular base strip, lower column, and pedestal. The next step involves using lifting equipment to position the rectangular base strip and the steel baseplate, after which the bolts are tightened to the specified torque. Then, the lower column and pedestal are placed, and after positioning the steel baseplate, the bolts are tightened according to the required torque. Following this, the upper column is placed, aligned with the flange plate, and high-strength bolts are tightened. Finally, soil backfilling and compaction are carried out to complete the process.

### 2.2 Establishment of a mortise-tenon and joint-flange assembled foundation model

#### 2.2.1 Element selection and mesh generation for assembled foundation models.

This study establishes a three-dimensional finite element model (FEM) of a assembled foundation using finite element analysis software and actual foundation parameters. The foundation’s main body is made of reinforced concrete. Considering the complexity and abundance of rebar types in actual structures, the rebars and concrete are modeled as separate entities in the 3D FEM. Concrete is represented using C3D8R solid elements, while rebars, being slender materials, are modeled using T3D2 truss elements. To accurately simulate the actual stress conditions of the combined rebar and concrete elements, the “Embedded” constraint feature in finite element software is typically used to bind the rebar and concrete elements as a whole [19]. Both the bolts and joint-flanges are made of steel. To better simulate their interaction with concrete, the same C3D8R solid elements used for the concrete structure are selected for these components.

During the mesh generation process, the mesh density directly affects the accuracy and computational time of the model. Considering that the primary research focus is the concrete foundation, the mesh sizes for concrete and rebar elements need to be more refined, with mesh densities set to 0.02 and 0.01, respectively. For the joint-flange plate and bolt elements, which are not the primary focus of the study, the mesh sizes can be slightly increased to reduce computational time, with mesh densities set to 0.03 and 0.04, respectively.

**Fig 3:**
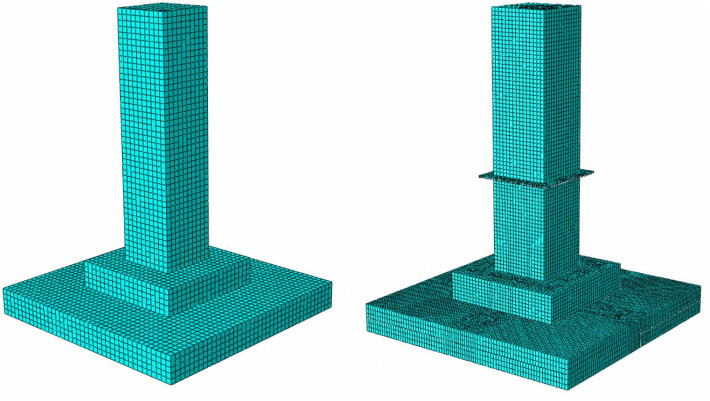
Finte element model of the cast-in-place foundation and the assembled foundation.

To study the deformation state and stress distribution of the MTJF assembled foundation compared to cast-in-place foundations, a cast-in-place concrete foundation of the same size and specifications was established following the aforementioned settings. In the numerical model of this study, each component, such as the precast slabs, is modeled individually and then assembled together. The contact is set as a conventional contact, with the tangential behavior defined by a “penalty function” and the normal behavior by “hard contact” with “separation allowed after contact.” It is important to note that in order to simulate the effect of high-strength bolts, the bolts and nuts are modeled as a unified entity to simulate their connecting function under normal working conditions. At the same time, the soil model uses the Mohr-Coulomb model, which can correctly simulate the behavior of the soil after the interaction between the foundation and the soil. The finite element model of the foundation is shown in Fig 3.

#### 2.2.2 Configuration of simulated materials.

The materials used for simulating the foundation are primarily divided into concrete and steel materials. According to the requirements outlined in the “Code for Design of Concrete Structures” [20], the concrete strength of assembled foundations must not be lower than C25. High-strength bolts of grade 8.8 M20 are selected for the connecting bolts, HRB400 grade hot-rolled ribbed steel bars are used for reinforcement, and Q345 steel is employed for the steel structure joint-flanges. The concrete material employs a damage-plasticity model, simulating its elastoplastic behavior under actual loading by configuring parameters such as elastic modulus and expansion angle. The steel structural materials adopt an ideal plastic constitutive model to simplify computational time. This assumes that after yielding under load, the stress-strain curve in the plastic phase continues to exhibit an elastic trend. The corresponding material parameters are presented in Table 2.

**Table 2:** Physical properties of materials.

	Density $\text{\textbackslash{}bf ×\textbackslash{} }10^{-9}/(t/mm^{3})\backslash )$	Elastic Modulu/MPa\)	Poisson’s Ratio	Expansion Angle/(°)	Eccentricity	Viscous Parameters	Yield Stress /MPa\)
C25 Concrete	2.4	28 000	0.2	30	0.1	0.005	–
Connecting Bolts	7.8	210 000	0.3	–	–	–	360
Reinforcing Steel	7.8	210 000	0.3	–	–	–	270
Joint-fange	7.8	210 000	0.3	–	–	–	360

### 2.3 Load conditions and loading methods

#### 2.3.1 Uplift load-horizontal load simulation.

When conducting foundation design and bearing capacity studies, it is important to recognize that combined vertical and horizontal loads are the primary controlling loads. Therefore, the loading conditions in the numerical simulation process are categorized into two types: uplift-horizontal loads and downward-horizontal loads. Considering the typical issue of inadequate uplift bearing capacity in concrete foundations, a static analysis of both assembled and cast-in-place foundations is conducted using uplift-horizontal combined loading conditions.

When analyzing the stress characteristics and deformation trends of the new type of concrete assembled foundation, it is necessary for the foundation to operate continuously under normal conditions for a period of time. Therefore, the design value of the foundation’s acting force is selected as the loading magnitude, and the effect of the foundation continuously bearing the load transmitted from the superstructure over time is simulated by defining the loading amplitude curve as an “instantaneous load” in the finite element analysis software. However, when investigating the bearing characteristics of the new type of concrete assembled foundation, it is necessary to continuously load the foundation until it fails. According to the Reference [21] sustained load method proposed in reference, the load increment rate for each level is approximately 10% of the anticipated ultimate load. This stepwise loading approach, as conducted in the literature experiments, is implemented by defining the loading amplitude as a “uniformly increasing curve” in the finite element analysis software. Referring to the “Design Code for Transmission Line Towers and Foundations” for solving the acting forces on foundations and combining it with the actual geological parameters of the new type of assembled foundation, the design acting forces for uplift and horizontal directions under the most unfavorable conditions are determined to be 120 kN and 35 kN, respectively. Additionally, referencing studies [22–24] on the ultimate bearing capacity of foundations under combined loading conditions from references, the anticipated ultimate bearing capacities in the uplift and horizontal directions in this study are 800kN and 100kN.

#### 2.3.2 Foundation soil pressure simulation.

Under combined uplift and horizontal loads, according to the foundation’s ultimate uplift bearing capacity calculation formulas (1) and (2) [25], the ultimate uplift bearing capacity of the foundation is jointly determined by the foundation’s self-weight and the weight of the foundation soil. In other words, the foundation resists the destructive uplift force through the downward pressure exerted by the foundation soil on the upper surface of the foundation base and the foundation’s own weight:


$$T=\gamma_{E}\gamma_{s}\gamma_{\theta_{1}}V_{t}+G$$ (1)

$$V_{t}=h_{t}\left(B^{2}+2Bh_{t}\tan\alpha -\frac{4}{3}h_{t}^{2}\tan^{2}\alpha \right)$$ (2)

Where, $\gamma_{E}$ is the horizontal force influence coefficient; $\gamma_{s}$ is the unit weight of the soil; $\gamma_{\theta_{1}}$ is the volume of soil that covers the foundation and resists uplift forces; G is the weight of the foundation itself, which contributes to resisting uplift forces; $h_{t}$ is the depth at which the foundation is buried into the soil, influencing the foundation’s stability and bearing capacity; α is the angle at which soil uplift forces are applied to the foundation, affecting the foundation’s resistance to uplift.

To accurately calculate the ultimate bearing capacity of the foundation under corresponding geological conditions, pressure is applied to the upper surface of the base in the foundation’s finite element model to simulate the downward pressure exerted by the foundation soil. The required pressure magnitudes, calculated based on different geological condition parameters, are shown in Table 3. Since the actual engineering environment for the new concrete assembled foundation predominantly consists of dense clay, a uniform pressure of 50 MPa is applied to the rectangular base of the foundation to simulate the downward pressure exerted by the foundation soil.

**Table 3:** Simulation of soil pressure under different geological conditions.

Geological Environment	Clay and Silty Clay (Hard)	Clay and Silty Clay (Soft Plastic)	Silt (Dense)	Silt (Slightly Dense)	Sandy Soil (Gravelly Sand)	Sandy Soil (Fine Sand)
Bulk Density	17	15	17	15	19	16
Uplift Angle	20	10	25	15	30	26
Applied Pressure	47.02	21.25	47.42	31.06	102.52	69.42

### 2.4 Experimental verification

#### 2.4.1 Overview of the experiment.

The experiment site is located in a rural area of Zhumadian, Henan Province. The altitude of the site area is approximately 82m. The stratum that is widely distributed in the area and has a certain thickness is the silt – gravel – containing soil layer, and some tower foundations are directly located within this layer. Based on the on-site exploration data and indoor geotechnical experiments, it can be known that the lithology of the strata within the exploration scope of the experiment site is relatively simple, mainly divided into the Quaternary silty gravel layer and silty sand – gravel layer. Samples were taken from the two types of foundation soils exposed by the boreholes. In the upper soil layer, the gravel content is greater than 50%, the content of fine gravel less than 0.075 mm is 15.56%, the silt particle content is greater than the clay particle content, the coefficient of non-uniformity (*C*_*u*_) is 79, and the coefficient of curvature (*C*_*c*_) is 1.034. It belongs to well – graded silt soil. In the lower soil layer, the particle – size composition is mainly sandy soil. The content of fine – grained particles less than 0.075 mm is 30.13%, the silt particle content is greater than the clay particle content, with (*C*_*u*_)= 89 and ((*C*_*c*_)=0.625. It belongs to poorly – graded silty sand.

The physical and mechanical parameters of different soil layers in the site are shown in Table 4. With the change of depth, the change degree of the basic physical properties of the foundation soil is not significant, and there is a certain spatial difference in physical and mechanical properties. The cohesion of the silty gravel at a depth of 0 ~ 3m ranges from 12.50 to 14.89kPa, and the internal friction angle is from 25.6° to 28.45°. With the increase of depth, the shear strength parameters show a weak increasing trend. There are significant differences in the strength parameters between the silty sand layer at a depth of 3 ~ 8m and the silty gravel. The cohesion is relatively large, with an average value of 22.04kPa, and the internal friction angle is relatively small, with an average value of 16.46°. Field investigations and drilling reveal that the groundwater depth in the site area is relatively large, with a depth greater than 10 m.

**Table 4:** Physical and mechanical parameters of soil layersat the experiment site.

Sample Number	Sampling Depth/m	Water Content/%	Compression Modulus/MPa	Cohesion/kPa	Internal Friction Angle/(°)
1−1	0 ~ 3.0	22.94	14.90	12.50	25.60
1-2	0 ~ 3.0	20.12	15.30	14.89	28.45
2−1	3.0 ~ 3.9	19.89	18.80	20.47	15.05
3−1	5.2 ~ 6.2	22.18	19.40	23.08	17.39
3−2	5.2 ~ 6.2	21.86	21.50	23.61	17.87
4−1	8.0 ~ 9.0	24.43	15.40	17.18	30.49

**Fig 4:**
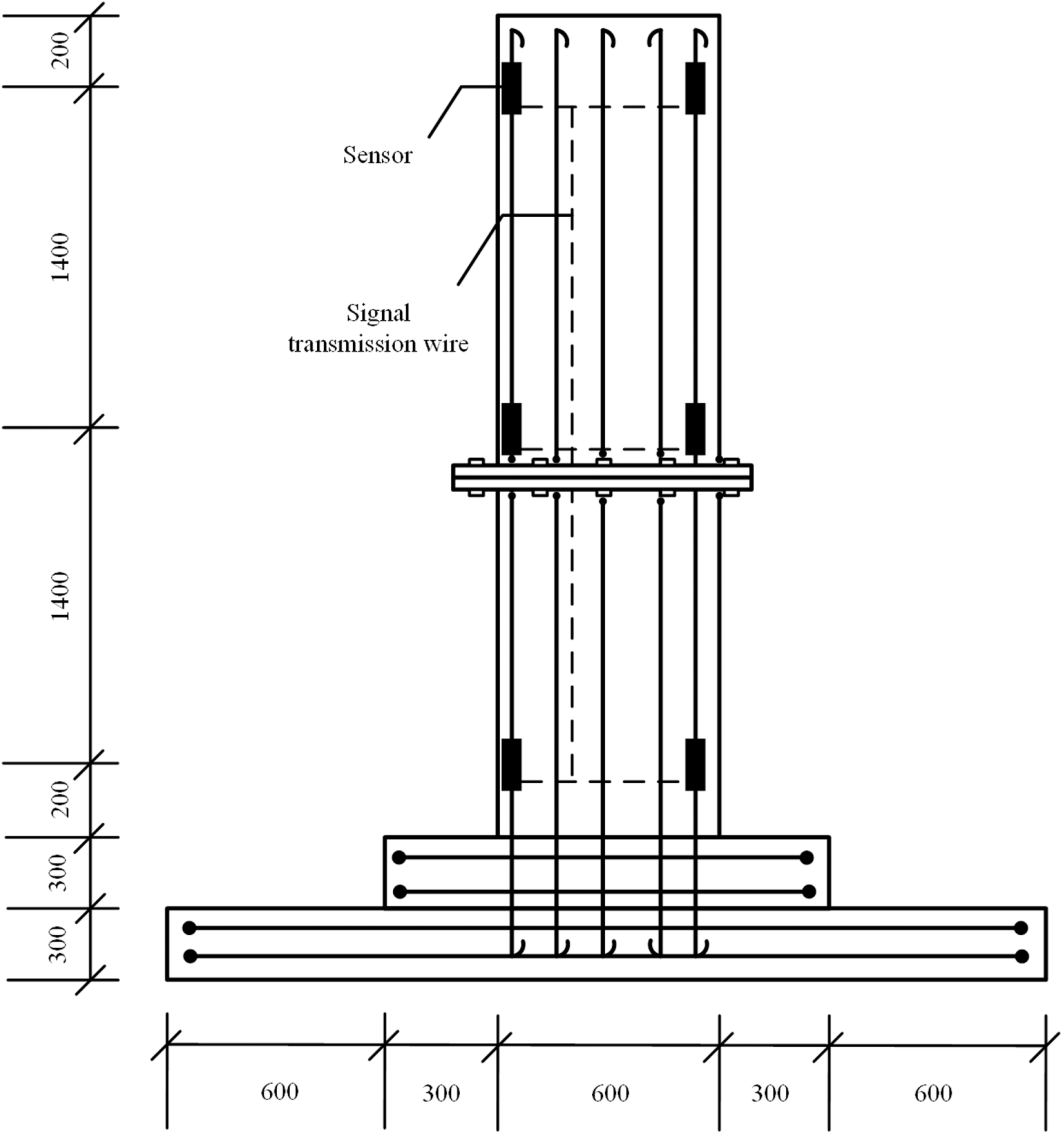
Sensor layout.

To verify the differences between numerical simulation and field experiments, uplift and horizontal load experiments were carried out on the MTJF assembled foundation for the experiment. Each MtJF assembled foundation consists of a bottom plate, a pedestal, two flange plates, and two columns, which are cast integrally. The dimensions of the column are 0.6m × 0.6m × 3.2m, the dimensions of the pedestal are 1.2m × 1.2m × 0.3m, and the dimensions of the bottom plate are 2.4m × 2.4m × 0.3m. Due to the limitation of experimental funds, six steel bar stress gauges were symmetrically arranged at different embedded depths of the column (200 mm below the column top, at the mid-height of the column, and 200 mm above the pile bottom) to ensure that the axial forces at the top, middle, and bottom could be monitored. At the same time, at least one pair of steel bar stress gauges was arranged in each soil layer of the pile body to monitor the axial force distribution and variation of the pile body in different soil layers. The specific layout is shown in Fig 4.

**Fig 5:**
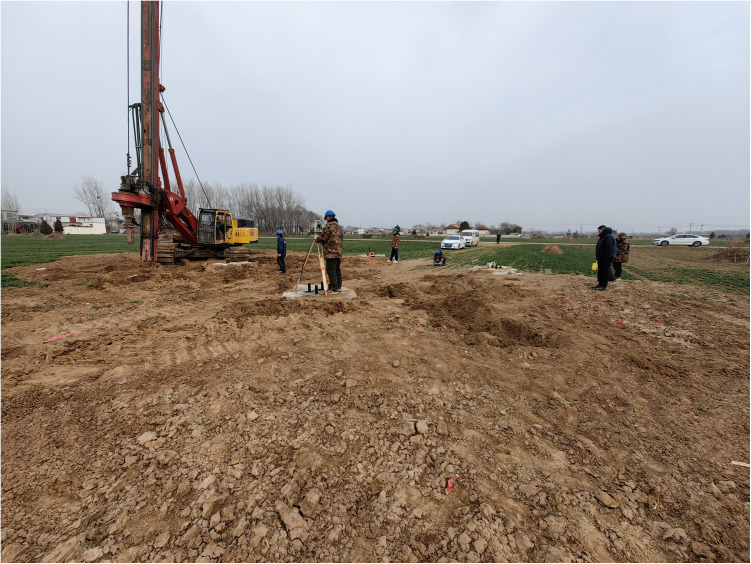
Drilling rig construction.

**Fig 6:**
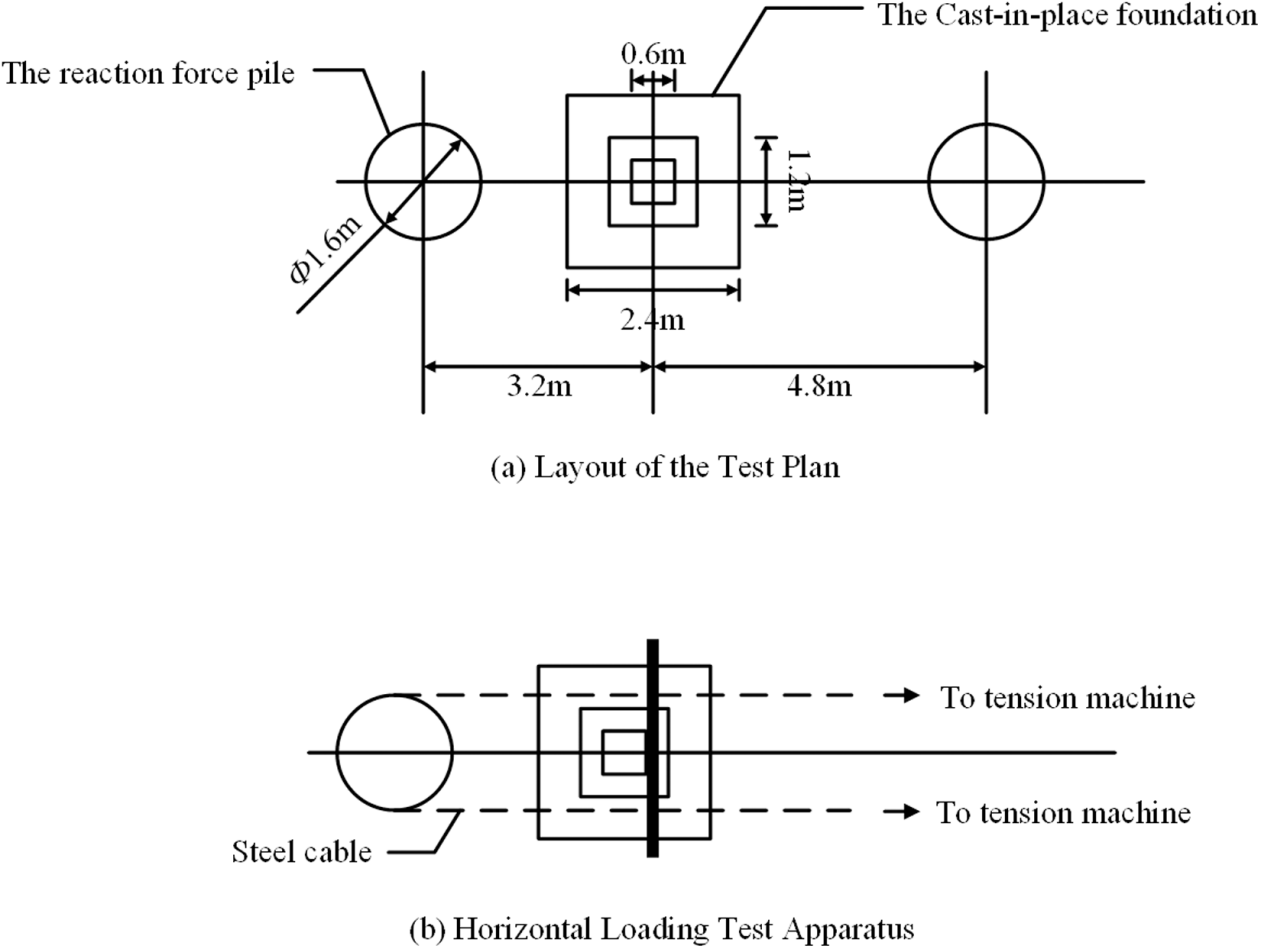
Experiment loading plan layout.

The experiment is provided with support reaction forces by the reaction force piles with a diameter of 1.6m and a length of 8m. The strength grade of the concrete used for the pile body in the experiment is C35.The reaction force piles were cast by excavating holes and pouring with a small-sized rotary drilling rig as shown in Fig 5. The two types of experiment loading devices and the on-site layout are shown in Fig 6.

**Fig 7:**
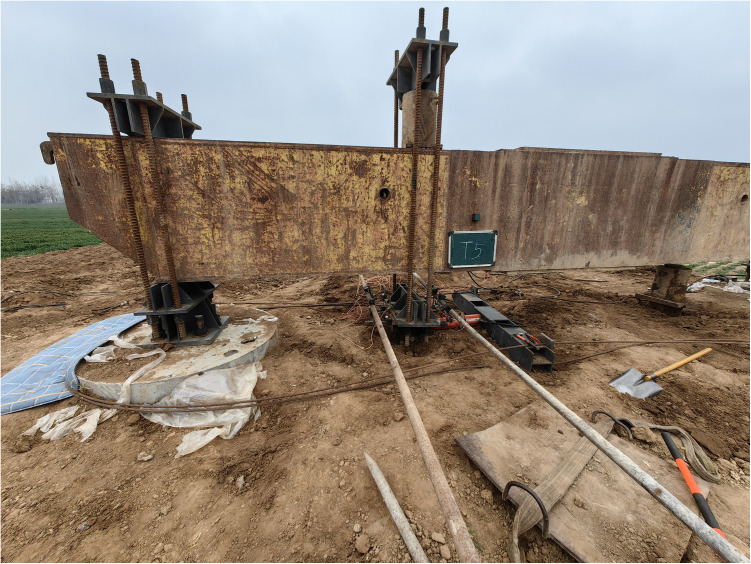
Experiment site.

The experiment loads the foundation according to the slow-maintained load method specified in the “Technical Code for Testing of Building Foundation Piles” (JGJ106–2014). The uplift and horizontal loads are applied in 8 steps, with each step load being 200 kN and 150 kN respectively. During the experiment, the experiment is terminated when the load causes the foundation to fail. It is specified that the load corresponding to the starting point of the steep drop or the settlement displacement reaching 30 mm is taken as the failure criterion. In the horizontal direction, the horizontal load corresponding to the displacement of the pile at the ground surface being 10 mm (6 mm for buildings sensitive to horizontal displacement) is usually taken as the horizontal ultimate bearing capacity of the pile. Due to the relatively small size of the pile used in this experiment, during step-by-step loading, each step of the uplift and horizontal loads is 20 kN and 15 kN.The experiment results show that the ultimate loads of the MTJF assembled foundation in two directions are 580 kN (uplift resistance) and 90 kN (horizontal resistance) respectively. The pictures of the field experiment are shown in Fig 7.

## 3. Results and analysis

### 3.1 Model validation

Taking the assembled foundation mortise-tenon joint-flange connector as the research object, the validity of the finite element simulation method designed in this study is demonstrated by comparing the error between the theoretical numerical calculation results and the finite element analysis results for the maximum stress on the joint-flange.

The calculation formula for the maximum tensile stress [26] that may occur on the surface of the joint-flange plate without stiffening ribs is shown in Formula (3),:


$$\sigma =\frac{5R_{f}e_{0}}{St^{2}}$$ (3)

$$e_{0}=\frac{1}{2}a$$ (4)

Where $R_{f}$ is the reaction force between the end joint-flange plates, $e_{0}$ is the bending moment acting on the joint-flange plate, S is the bolt spacing, t is the joint-flange plate thickness, and a is the vertical distance from the end of the joint-flange plate to the bolt. The joint-flange plate reaction force $R_{f}$ is calculated using Formula (5):


$$R_{f}=N_{b}\frac{b}{a}$$ (5)

$$b=\frac{1}{2}S$$ (6)

Where $N_{b}$ is the tensile force exerted on the joint-flange plate by the longitudinal reinforcement of the column, b is the vertical distance from the longitudinal reinforcement to the bolt. Based on the parameters derived from the dimensions of the new assembled concrete foundation structure and the magnitude of the simulated load, combined with Formulas (3)~(6), the maximum tensile stress $\sigma_{\max}$ that may occur on the joint-flange plate can be determined.

In this study, the predicted ultimate uplift capacity is 800 kN, and it can be assumed that the tensile force generated by the vertical reinforcement of the column on the flange plate is 800 kN at the ultimate state. Therefore, $N_{b}$ is taken as 800,000 N. According to formulas (4) and (5), e0 and a are located above and below the semicolon, respectively, and can be directly simplified, with the denominator remaining 2. Similarly, according to formulas (3), (5), and (6), b and s are also located above and below the semicolon, so they can be directly simplified, with the denominator remaining 2. The thickness of the flange plate in this study is 60 mm, and t is taken as 60. Combining these calculation conditions, the maximum tensile stress of the joint-flange plate is calculated to be $\sigma_{\max}$= 272.01 MPa.

**Fig 8:**
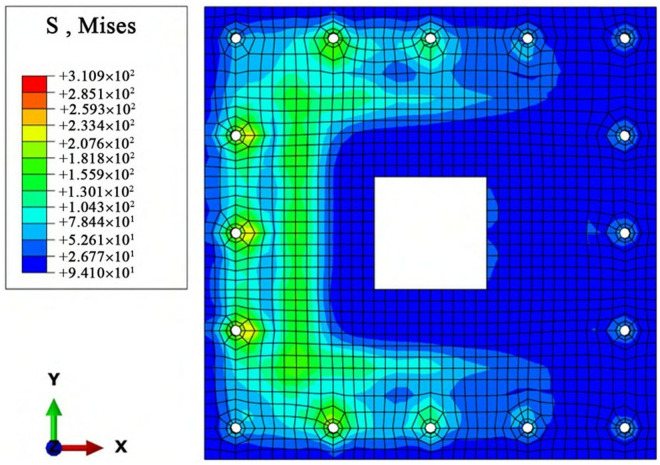
Stress contour map of the joint-flange plate.

The stress contour map of the joint-flange plate obtained through finite element analysis is shown in Fig 8.

From Fig 8, the maximum tensile stress of the joint-flange plate calculated by the finite element method is $\sigma_{\max}$=310.93MPa.

The stress on the joint-flange plate is influenced by the combined effects of bolts, longitudinal reinforcement, and concrete, resulting in relatively complex stress conditions. Therefore, the maximum stress error is relatively large, at 14.31%. However, the relative error between the maximum stress obtained from finite element analysis and theoretical calculation is within the allowable range of 15%. This indicates that the finite element simulation method for the new assembled concrete foundation proposed in this study is feasible, and the analytical data derived from the simulation model is reliable.

### 3.2 Stress analysis of foundations under combined loads

**Fig 9:**
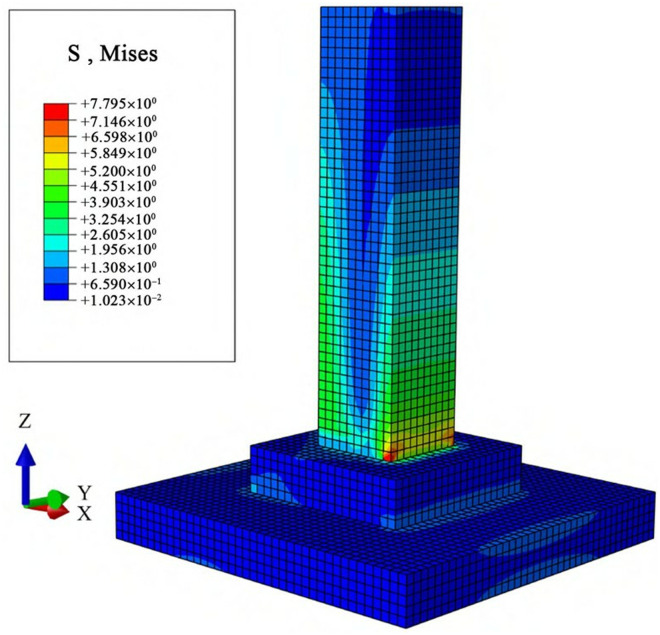
Overall stress cloud diagram of the cast-in-place foundation.

**Fig 10:**
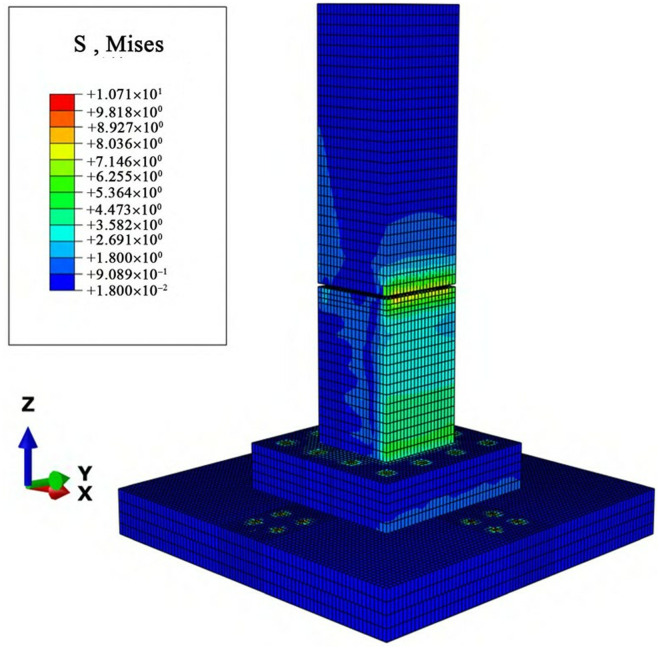
Overall stress cloud diagram of the assembled foundation.

A concentrated load is applied to the top surface of the foundation and uniformly distributed over the surface using the “point-to-surface coupling” method. The load magnitude is set according to the design values of the uplift and horizontal forces on the foundation. The overall stress distribution of the foundation (in MPa) is shown in the cloud diagram in Figs 9 and 10.

As shown in Figs 9 and 10, under the action of the uplift-horizontal combined load, the concrete stress of the typical slab-type cast-in-place foundation is mainly concentrated in the main column area. Large areas of stress concentration appear on both sides of the main column, with a distinct phenomenon of continuous stratification. The overall stress of the foundation varies with the height of the main column; the closer to the upper part of the main column, the more uniform the stress distribution and the smaller the stress. Conversely, the stress distribution becomes more concentrated and the stress increases. The maximum stress occurs at the location of the maximum bending moment on the column, which is at the bottom of the main column in the direction of the horizontal load.

The stress of the MTJF assembled foundation is mainly concentrated at the mortise-tenon and joint-flange connection joints and around the bolt holes on the pedestal and base. The column itself does not exhibit the stratified stress distribution observed in the cast-in-place foundation. This is because the mortise-tenon and joint-flange connection at the node within the column bears the main load transmitted from the upper column, resulting in stress concentration. Before the connection fails, the load borne by the corresponding location in the lower column is lower than that in the cast-in-place foundation. The maximum stress occurs at the mortise-tenon and joint-flange connection joint in the column in the direction of the horizontal load.

In summary, under the action of the uplift-horizontal combined load, the MTJF assembled foundation exhibits distinct stress characteristics compared to the cast-in-place foundation: **(1)** From the perspective of stress magnitude, the MTJF assembled foundation experiences slightly higher stress than the cast-in-place foundation, with the maximum stress occurring at the node within the column rather than at the point of maximum bending moment. **(2)** The MTJF assembled foundation exhibits a distinct stress distribution only in the direction of the horizontal load, with one side of the column bearing more significant stress. **(3)** When the upper load is transferred to the node within the column, the mortise-tenon and joint-flange connection bears the majority of the load, resulting in a more concentrated but lower stress distribution in the lower column compared to the cast-in-place foundation.

### 3.3 Displacement analysis of the foundation under combined loads

**Fig 11:**
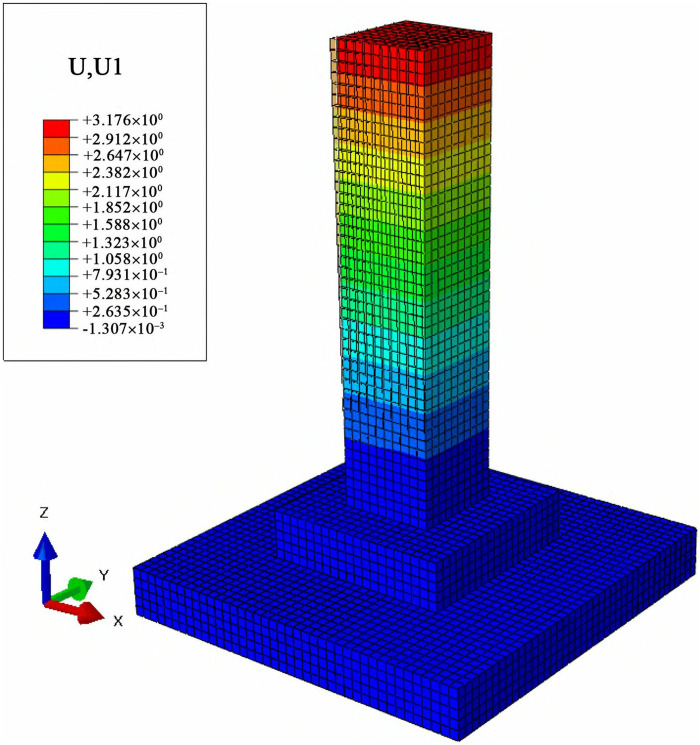
Horizontal displacement contour map of the Cast-in-place foundation (unit: mm).

**Fig 12:**
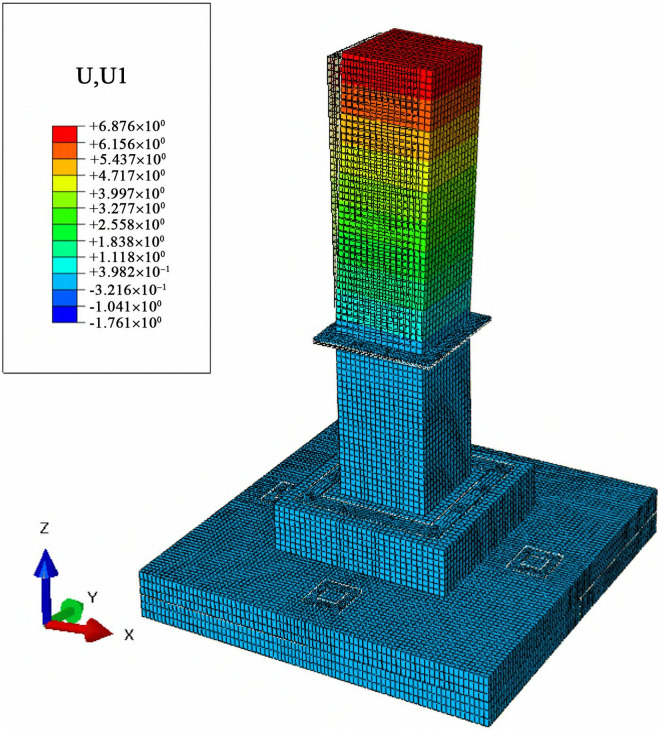
Horizontal displacement contour map of the assembled foundation (unit: mm).

**Fig 13:**
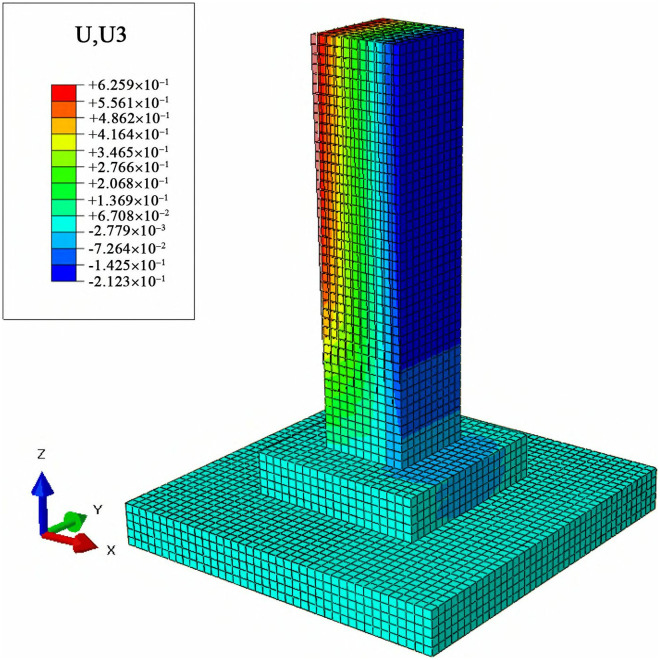
Vertical displacement contour map of the Cast-in-place foundation (unit: mm).

**Fig 14:**
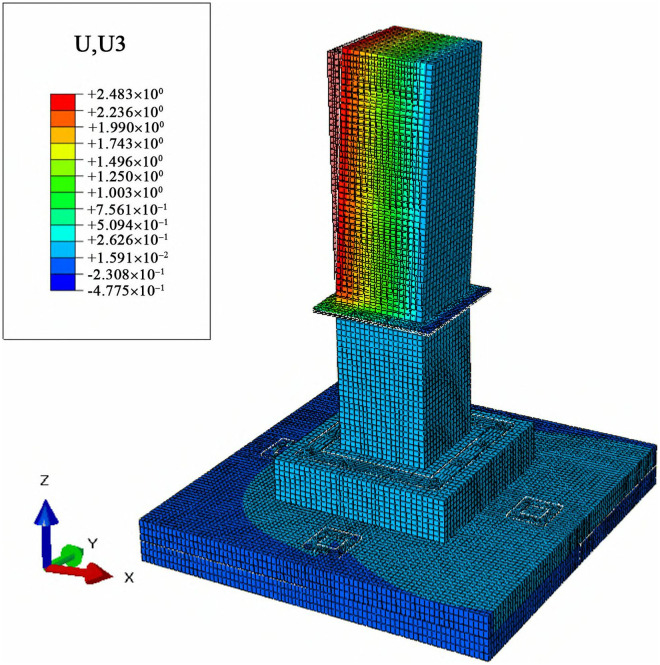
Vertical displacement contour map of the assembled foundation (unit: mm).

To analyze the lateral and vertical deformation of cast-in-place foundations and MTJF assembled foundation under uplift-horizontal combined loads, displacement contour maps along the horizontal load direction (X-axis) and vertical direction (Z-axis) were extracted. The results are shown in Figs 11–14.

#### 3.3.1 Displacement analysis of the foundation along the horizontal load direction under combined loads.

From Figs 11 and 12, it can be seen that under the uplift-horizontal combined loads, the horizontal displacement of the cast-in-place foundation mainly occurs in the column. The displacement gradually decreases from the top to the bottom of the column and exhibits a highly regular layered variation. The maximum horizontal displacement occurs at the top of the main column, approximately 3 mm. The horizontal displacement at the stepped base of the cast-in-place foundation under the combined loads is about 1/12 of the maximum horizontal displacement at the top of the column.

The deformation of the MTJF assembled foundation can be divided into two parts: The first part is a regular layered variation similar to that of the cast-in-place foundation. This deformation mainly occurs in the upper column of the foundation, with its horizontal displacement slightly larger than that of the cast-in-place foundation. Moreover, the horizontal displacement decreases approximately uniformly from the top of the upper column toward the middle node of the column. The second part is an overall uniform deformation without layered characteristics. This deformation occurs in the lower column of the foundation, the pedestal, and the base, where the horizontal displacement is relatively uniform, manifesting as overall deformation.

In summary, under the uplift-horizontal combined loads, the MTJF assembled foundation exhibits the following horizontal deformation characteristics: **(1)** The horizontal deformation of the MTJF assembled foundation is divided into two parts. The deformation of the upper column shows a layered pattern similar to that of the cast-in-place foundation and is more significant. However, the deformation from the middle node of the column downward to the pedestal differs greatly from the cast-in-place foundation, exhibiting characteristics of overall displacement deformation. **(2)** Due to the high elastic modulus of steel, the horizontal displacement of the joint-flange plate at the joint connection of the MTJF assembled foundation is lower than the displacement of the concrete at the joint. **(3)** The horizontal displacement from the foundation pedestal to the base is approximately 1/15 of the maximum displacement at the top surface of the main column. This indicates that during normal operation of the foundation, the horizontal displacement of the base is much smaller than that of the main column.

#### 3.3.2 Vertical displacement analysis of the foundation under combined loads.

From Figs 13 and 14, it can be observed that under the uplift-horizontal combined loads, the vertical displacement of the cast-in-place foundation primarily occurs in the main column. The distribution of vertical displacement from the left side to the right side of the main column shows a continuous layered arrangement. The displacement on the left side of the column is vertically downward, while the displacement on the right side is vertically upward. This indicates that under the combined loads, one side of the cast-in-place foundation is primarily under compression, while the other side is primarily under tension. The vertical displacement on the pedestal and the base is much smaller compared to the column and is uniformly in the downward direction.

The vertical displacement of the MTJF assembled foundation primarily occurs in the upper column. The distribution of vertical displacement from the left side to the right side of the upper column is similar to that of a typical slab-type cast-in-place foundation column, exhibiting a continuous layered arrangement and following a similar displacement direction pattern. The vertical displacement of the lower column and pedestal can be roughly divided into three parts: a large downward deformation region in the upper-middle part of the left side of the lower column, with a displacement of approximately 0.17 mm; a small downward deformation region in the middle part of the lower column and the left majority of the pedestal, with a deformation magnitude only 1/8 that of the large deformation region; and an upward deformation region on the right small portion of the lower column and the right half of the pedestal, where the deformation magnitude is 1–2 times that of the large downward deformation region. In addition, other parts of the base exhibit varying degrees of vertical downward deformation, but the deformation magnitude is relatively small.

In summary, under the uplift-horizontal combined loads, the MTJF assembled foundation exhibits the following vertical deformation characteristics: **(1)** Similar to the characteristics of horizontal deformation, vertical deformation of the foundation is also divided by the middle node of the column. The vertical deformation of the upper column has the same characteristics as that of the cast-in-place foundation, with one side under tensile deformation and the other side under compressive deformation. **(2)** The vertical deformation of the lower column and pedestal is divided into three regions: a large downward deformation region, a small downward deformation region, and an upward deformation region. The small downward deformation region accounts for 2/3 of the total deformation area, while the deformation magnitudes of the large downward deformation region and the upward deformation region are approximately 8 times and 16 times that of the small downward deformation region, respectively. **(3)** Similar to horizontal deformation, during normal operation of the foundation, the vertical displacement of the base is much smaller than that of the main column.

### 3.4 Ultimate bearing capacity analysis and experimental verification of the foundation under combined loads

#### 3.4.1 Ultimate uplift bearing capacity analysis and experimental verification of foundations under combined loads.

**Fig 15:**
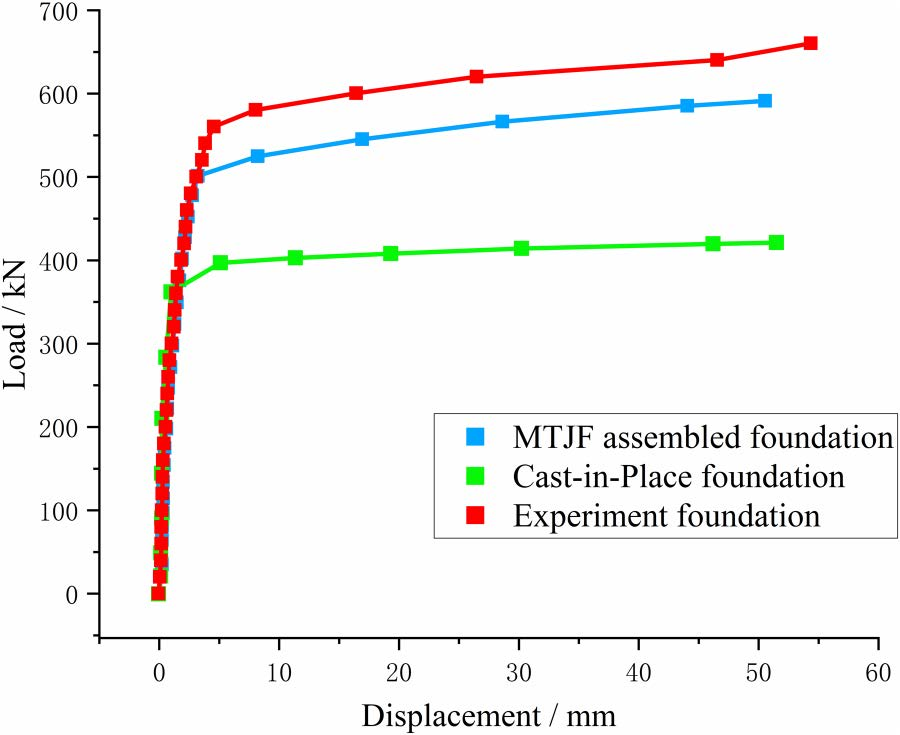
Uplift load-displacement relationship curve of the foundation.

**Fig 16:**
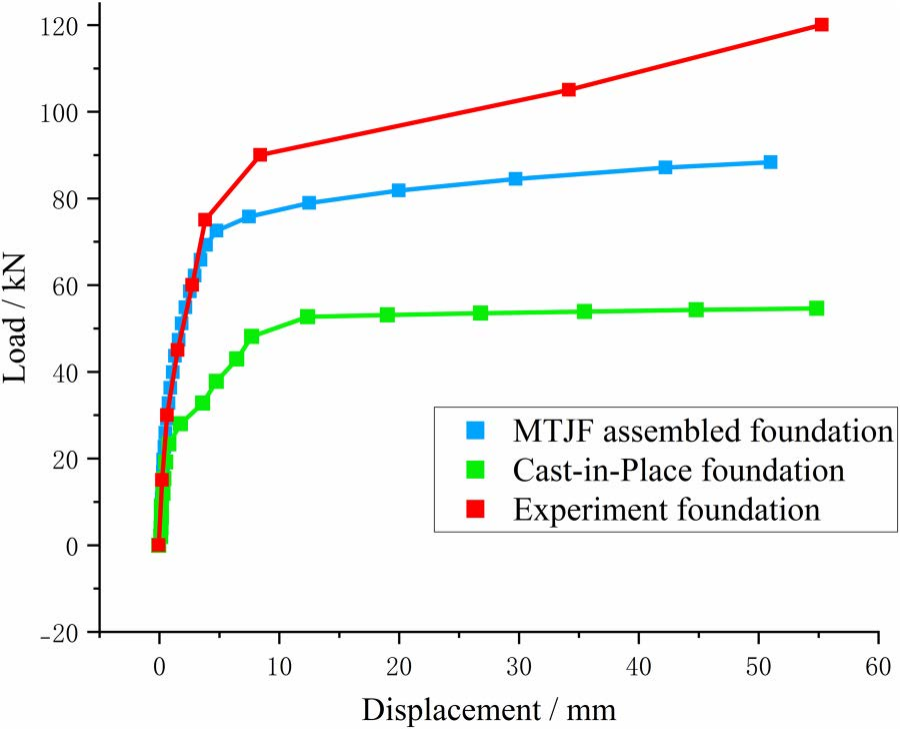
Horizontal load-displacement relationship curve of the foundation.

A concentrated load was applied to the simulated top surface of the foundation, and the load was uniformly distributed over the foundation’s top surface using the “point-to-surface coupling” method. The load magnitude was set to the ultimate bearing capacity in the uplift and horizontal directions. The displacement-load curves for the new assembled concrete foundation and the cast-in-place foundation are shown in Figs 15 and 16.

Numerical simulation calculations were carried out on the foundations under the two loading methods of the field experiment. By comparing the numerical simulation calculation results with the experiment results, it can be known that the simulated ultimate bearing capacity is slightly less than the experiment result.

When the uplift load reaches 580 kN, there is a mutation point on the load-displacement curve of the MTJF assembled foundation, indicating that relative slip occurs between the pile and the soil at this time. Therefore, for the ultimate bearing capacity of the MTJF assembled foundation calculated by numerical simulation, the load values corresponding to the standard failure displacement specified in the code are taken. The main reasons for analysis are as follows:

(1) In the numerical simulation, the voids between the adjacent structures of the pile and the soil are simulated by assigning stiffness to the contact elements, and it is assumed that the contact surface stiffness remains unchanged in the elastic stage, which leads to a larger simulated result. However, in reality, the “contact stiffness” between the pile and the soil decreases with the application of the load.
(2) In the field experiment, there are generally soil disturbances and errors in the process of excavating and pouring the reaction force piles and the experiment piles.

However, in summary, the numerical simulation results were compared with the experimental data to evaluate the accuracy of the model. In the experiment, the ultimate uplift load of the Experiment Foundation was 580 kN. In the numerical simulation, the MTJF foundation exhibited an ultimate uplift load of 540 kN, with an error of 7.41%.The results indicate a good agreement between the numerical simulation and experimental ultimate load, suggesting that the numerical model accurately reflects the actual working condition of the foundation.

From Fig 15, it can be seen that under the uplift-horizontal combined loads, when the uplift force on the cast-in-place foundation is less than 350 kN, the foundation exhibits an elastic deformation trend, with a slow rate of increase in vertical displacement. The uplift force and vertical displacement show a nearly linear relationship. When the uplift force increases to 405 kN, the rate of displacement growth starts to increase, and the foundation quickly enters a state of linear instability. At this ultimate state, the cumulative upward displacement at the top of the foundation reaches 9.5 mm. The vertical displacement of the MTJF assembled foundation is similar to that of the cast-in-place foundation. When the uplift force is no greater than 500 kN, the foundation exhibits elastic deformation. Beyond this value, plastic failure occurs in the weaker parts of the foundation, and the rate of vertical displacement change gradually accelerates. When the uplift force reaches 540 kN, the load-displacement curve approaches a linear trend, and the total upward displacement of the foundation is 13.8 mm.

According to the “Technical Specifications for Building Pile Testing” (JGJ106–2014), the failure criterion is defined as the point where the load or settlement displacement reaches 30 mm, with the corresponding uplift and downward load taken as the ultimate uplift and downward bearing capacity of the pile. From Fig 15, the load corresponding to the start of the steep drop for the cast-in-place foundation is 405 kN, with a cumulative displacement of 9.5 mm at the foundation surface when the ultimate limit state is reached. Based on the given working conditions in this study, we define the ultimate uplift load of the cast-in-place foundation as 405 kN. In contrast, for the mortise-flange prefabricated foundation, the load at the start of the steep drop is 540 kN, with a cumulative displacement of 13.8 mm at the foundation surface when the ultimate limit state is reached. Under these conditions, the ultimate uplift load for the mortise-flange prefabricated foundation is defined as 540 kN. Compared to the cast-in-place foundation, the cumulative displacement of the foundation surface for the mortise-flange prefabricated foundation is nearly identical at the same uplift load. More notably, the ultimate uplift load of the mortise-flange prefabricated foundation shows a significant improvement, increasing by 33.34% compared to the cast-in-place foundation.

#### 3.4.2 Ultimate horizontal bearing capacity analysis and experimental verification of foundations under combined loads.

As shown in Fig 16, when the horizontal load reaches 90 kN, a mutation point appears on the load-displacement curve of the MTJF assembled foundation, indicating relative slip between the pile and the soil. In the numerical simulation, the MTJF foundation demonstrated an ultimate horizontal load of 77 kN, with an error of 16.88%. The simulated ultimate bearing capacity is slightly less than the experiment result.

Aside from reasons similar to those mentioned above, another cause is that in the horizontal loading experiment, the middle part of the steel wire rope is connected to the flange plate. Due to the loss of force transmission at the connection point, the growth of the horizontal displacement in the later stages of loading is relatively small.

Overall, as concluded earlier, the results show good agreement between the numerical simulation and the experimental ultimate load, indicating that the numerical model accurately reflects the actual working conditions of the foundation.

From Fig 16, the horizontal load-displacement relationship curves of the cast-in-place foundation and the MTJF assembled foundation are similar to the uplift load-displacement relationship curves. Under horizontal loading, the foundation also exhibits three stages: an elastic deformation stage, a plastic transition stage, and a linear failure stage. Specifically, the cast-in-place foundation reaches its ultimate state after a cumulative horizontal displacement of 12 mm, at which point the horizontal load is 52 kN. In contrast, the MTJF assembled foundation reaches its ultimate state after a cumulative displacement of 9.1 mm, with a corresponding horizontal load of 77 kN.

According to the “Technical Specifications for Building Pile Testing” (JGJ106–2014), in the horizontal direction, the horizontal load corresponding to a displacement of 10 mm at the pile-ground interface is commonly taken as the horizontal ultimate bearing capacity of the pile (for buildings sensitive to horizontal displacement, a value of 6 mm is used).

From Fig 16, the load corresponding to the start of the steep drop for the cast-in-place foundation is 52 kN, with a cumulative horizontal displacement of 12 mm at the foundation surface when the ultimate limit state is reached. Based on the given working conditions in this study, we define the ultimate uplift load of the cast-in-place foundation as 52 kN. In contrast, for the mortise-flange prefabricated foundation, the load at the start of the steep drop is 77 kN, with a cumulative horizontal displacement of 9.1 mm at the foundation surface when the ultimate limit state is reached. Under these conditions, the ultimate uplift load for the mortise-flange prefabricated foundation is defined as 77 kN. Compared to the cast-in-place foundation, the mortise-flange prefabricated foundation exhibits smaller horizontal displacement under the same horizontal load. In the plastic overstrain phase, the mortise-flange prefabricated foundation does not experience significant horizontal displacement, which is an advantage for towers that are sensitive to horizontal displacement. More notably, the ultimate horizontal uplift load of the mortise-flange prefabricated foundation shows a significant improvement, increasing by 48.09% compared to the cast-in-place foundation.

#### 3.4.3 Ultimate bearing capacity characteristics analysis of foundations under combined loads.

Comparing the load-displacement relationship curves of the two types of foundations, it can be observed that under uplift-horizontal combined loads, the relationship curve is divided into three distinct stages: **(1)** The elastic deformation stage, where the foundation as a whole undergoes elastic changes, showing a linear upward trend, and the top displacement of the foundation increases linearly and slowly as the uplift force increases. **(2)** The plastic transition stage, which occurs when the weak parts of the foundation begin to yield. During this stage, the top displacement of the foundation continues to increase with the applied uplift force, and the rate of increase accelerates. **(3)** The linear instability stage, which occurs when the foundation as a whole yields. At this stage, the top displacement of the foundation increases rapidly with the uplift force, and the foundation cracks at its weakest part, leading to complete failure and loss of normal functionality. Both types of foundations exhibit typical uplift behavior [27], indicating that the uplift performance of the MTJF assembled foundation is not substantially different from that of the cast-in-place foundation.

Whether it is the ultimate uplift load or the ultimate horizontal load, the MTJF assembled foundation demonstrates significantly superior ultimate bearing capacity compared to the cast-in-place concrete foundation. Moreover, when the MTJF assembled foundation reaches its ultimate state, the displacement-load relationship curve still shows a certain elastic tendency as a “slanted segment,” rather than the “linear segment” that indicates complete failure in the cast-in-place foundation. Based on the force characteristics of the two types of foundations, it is evident that the MTJF assembled foundation resists uplift-horizontal loads by having both the concrete upper column and the mortise-tenon and joint-flange connection joint share the load. Unlike the cast-in-place concrete foundation, which rapidly destabilizes and fails upon reaching its ultimate state, the MTJF assembled foundation transfers the load in stages. After the concrete upper column yields, the mortise-tenon and joint-flange connection joint in the steel-concrete structure takes on the load transferred by the upper column. Only when the displacement at the joint reaches its limit does the load continue to transfer to the lower column of the foundation. This segmented load transfer mechanism indicates that the MTJF assembled foundation possesses excellent bearing capacity.

## 4. Conclusion

Using finite element analysis software to simulate the bearing performance of the MTJF assembled foundation under uplift-horizontal combined loads, and based on the analysis and study of the numerical simulation results of the MTJF assembled foundation and cast-in-place foundation of the same specifications, the following main conclusions are drawn.

(1) Under combined loads, the deformation of the base in the MTJF assembled foundation is significantly smaller than that of the main column, indicating that the bearing capacity of the foundation base is not fully utilized. The maximum stress occurs at the mortise-tenon and joint-flange connection joint in the middle of the column.
(2) The uplift performance of the MTJF assembled foundation is not substantially different from that of the cast-in-place foundation. Its combined load-displacement relationship curve exhibits a similar pattern to that of the cast-in-place stepped foundation, showing typical uplift behavior that can be roughly divided into three stages: a slow linear increase stage, a plastic accelerated increase stage, and a linear failure stage.
(3) When the cumulative uplift displacement of the tenon-mortise-flange prefabricated foundation reaches approximately 13 mm and the cumulative horizontal displacement reaches around 10 mm, the foundation enters the ultimate state. Compared to the cast-in-place foundation of the same specification, the ultimate bearing capacity of this foundation is significantly enhanced, with the ultimate uplift bearing capacity increasing by 33.34% and the ultimate horizontal bearing capacity increasing by 48.09%. When the uplift limit state is reached, the displacement of the foundation is similar to that of the cast-in-place foundation; however, at the horizontal limit state, the horizontal displacement of the foundation is smaller than that of the cast-in-place foundation. Furthermore, this foundation offers distinct advantages in terms of transport and assembly, demonstrating substantial engineering application value.
(4) The MTJF assembled foundation exhibits a “segmented transfer” force characteristic. After the concrete upper column yields, the mortise-tenon and joint-flange connection joint takes on the load transferred by the upper column. Only when the displacement at the joint reaches its limit does the load continue to transfer to the lower column of the foundation. This force characteristic gives the MTJF assembled foundation excellent bearing capacity. Increasing the number of bolts at the mortise-tenon and joint-flange connection joint or optimizing the joint-flange parameters can effectively enhance the overall bearing capacity of the foundation.

## Supporting information

S1 Data Numerical values used to generate Fig 15. (XLSX)

S2 Data Numerical values used to generate Fig 16. (XLSX)
